# Fifty Years of JC Polyomavirus: A Brief Overview and Remaining Questions

**DOI:** 10.3390/v12090969

**Published:** 2020-09-01

**Authors:** Abigail L. Atkinson, Walter J. Atwood

**Affiliations:** Department of Molecular Biology, Cell Biology and Biochemistry, Brown University, Providence, RI 02912, USA; abigail_atkinson@alumni.brown.edu

**Keywords:** progressive multifocal leukoencephalopathy, JC polyomavirus, polyomavirus, HIV/AIDS, multiple sclerosis, autoimmune disease

## Abstract

In the fifty years since the discovery of JC polyomavirus (JCPyV), the body of research representing our collective knowledge on this virus has grown substantially. As the causative agent of progressive multifocal leukoencephalopathy (PML), an often fatal central nervous system disease, JCPyV remains enigmatic in its ability to live a dual lifestyle. In most individuals, JCPyV reproduces benignly in renal tissues, but in a subset of immunocompromised individuals, JCPyV undergoes rearrangement and begins lytic infection of the central nervous system, subsequently becoming highly debilitating—and in many cases, deadly. Understanding the mechanisms allowing this process to occur is vital to the development of new and more effective diagnosis and treatment options for those at risk of developing PML. Here, we discuss the current state of affairs with regards to JCPyV and PML; first summarizing the history of PML as a disease and then discussing current treatment options and the viral biology of JCPyV as we understand it. We highlight the foundational research published in recent years on PML and JCPyV and attempt to outline which next steps are most necessary to reduce the disease burden of PML in populations at risk.

## 1. Introduction

Viruses in the family Polyomaviridae are found in a wide range of host species [1,2,3,4,5,6]. Since the first polyomavirus was isolated in 1952 and identified as Murine K virus, 73 species have been identified, 13 of which are found in humans [7,8,9,10,11,12,13,14,15,16,17,18,19,20]. JC polyomavirus (JCPyV) and BK polyomavirus (BKPyV), the first human polyomaviruses to be discovered, were isolated in 1971 from patients whose initials were JC and BK, respectively [8,9]. Although epidemiological studies on JCPyV are sparse, it is clear both viruses are highly prevalent in the worldwide population. Seroprevalence falls between 40% and 60% for JCPyV and is greater than 80% for BKPyV [6,21,22,23,24,25]. JCPyV seroprevalence begins at a rate of 10–20% in childhood and increases steadily each decade, with about 50% of adults age 70 testing positive, though some research has estimated values even higher [23,24]. For the purposes of this review we will focus on JCPyV, which is the only human polyomavirus known to cause disease of the nervous system [26,27].

## 2. JCPyV-Induced Diseases

### 2.1. Progressive Multifocal Leukoencephalopathy (PML)

PML was first identified as a unique pathology in 1958 as a complication of primary B cell lymphoproliferative disorders [28,29,30,31]. All known PML cases at the time presented with multifocal white matter lesions in the central nervous system (CNS) accompanied by a triad of histopathological symptoms—demyelination, abnormal astrocytic morphology and oligodendrocytic nuclear inclusions [32]. The presence of inclusion bodies in oligodendrocytes suggested a viral cause [28,33,34]. PML remained a very rare disease until the mid-1980s, when acquired immunodeficiency syndrome (AIDS) reached epidemic and eventually pandemic proportions [35]. PML became an AIDS defining illness and 2–5% of HIV infected individuals would go on to develop this uniformly fatal disease [36,37,38,39,40]. The introduction of highly active antiretroviral therapy (HAART), a treatment that improves immune function through suppression of the HIV virus, led to a steep decline in AIDS-related PML fatalities, but did not eliminate AIDS-related PML entirely [41]. While the fatality rate of PML has been significantly reduced by restoration of immune function and most PML patients now survive, most experience significant morbidity [42,43,44,45]. HIV-associated PML still remains one of the most common types of PML, but no longer accounts for the overwhelming majority of new cases due to the availability of HAART and the emergence of new risk factors for PML [46,47]. Though it is one of the only treatments available for PML, restoration of immune function needs be carefully managed because it can lead to a fatal inflammatory reaction called immune reconstitution inflammatory syndrome or IRIS [48,49,50].

### 2.2. Drug-Induced PML

AIDS and lymphoproliferative disease are no longer the only major risk factors for PML. The relatively recent development of powerful immunomodulatory drugs for treating multiple sclerosis and other autoimmune disorders have led to a new population of PML-susceptible individuals. This group is primarily composed of individuals taking immunosuppressants like natalizumab, a monoclonal antibody designed to reduce trafficking of leukocytes into inflamed tissue [46,51,52,53]. Although this group has historically been smaller than those affected by HIV or cancers affecting the immune system, an expansion of immunosuppressive drugs in the last 15 years has led to a rapid increase in individuals affected with autoimmune treatment-associated PML. Certain immunosuppressants (most notably natalizumab, rituximab and dimethyl fumarate) still pose a significant risk for development of PML, depending on patient history and seropositivity for JCPyV [46,48,54,55,56,57].

Overall, PML associated with lymphoproliferative disorders, AIDS and immunomodulatory therapies make up an overwhelming majority of PML cases. PML is nearly universally associated with compromised cellular immunity. As such, other classifications of PML are exceedingly rare [58,59]. The prevalence of PML worldwide is often estimated to be about two cases per 100,000 individuals, though this number varies by population [47,60].

### 2.3. PML Symptoms and Treatment Options

For those individuals who develop PML, the effect is devastating. Once activated, JCPyV causes a lytic infection in oligodendrocytes and astrocytes in the CNS, affecting cells that are vital for neuronal stability [32]. Infection of oligodendrocytes leads to extensive demyelination resulting in neuronal dysfunction and death. Because demyelination occurs at distinct points, physical symptoms can mirror several different diagnoses, including multiple sclerosis and stroke. Such physical symptoms of PML are diverse and include motor dysfunction, visual defects and speech impairment [28,30,34]. Some individuals with PML may develop seizures as well [61]. While earlier diagnosis leads to better outcomes for patients, it is difficult to identify early stages of PML, meaning diagnosis often only occurs once symptoms are already severe [61,62]. A diagnosis of PML is made using initial presentation of neurological symptoms, magnetic resonance imaging (MRI) identification of contrast-enhanced viral lesions and confirmation of the presence of JCPyV DNA in the cerebrospinal fluid (CSF) of symptomatic patients [32,63]. PML lesions associated with different underlying disorders may present differently in MRI scans, and while diagnostic criteria for use in differentiating PML lesions from those caused by other diseases (such as multiple sclerosis (MS)) have been published, lesions remain difficult to diagnose particularly for individuals who are initially asymptomatic [32,64,65,66].

Immunosuppression is the major risk factor for development of PML [67]. More specifically, low CD4+ T cell response correlates with higher disease severity in HIV-positive individuals as well as in individuals with other immunosuppressive conditions [42,68,69,70,71]. CD8+ T cell response additionally appears to be important for recovery from PML; JCPyV-specific CD8+ T cells were found frequently in survivors of PML but were almost absent in patients with severe progressing PML [71,72]. This correlation between CD8+ T cells and a better recovery may be useful in treatment and prognosis, as it appears CD4+ T cells may primarily target more variable VP1 protein epitopes, while CD8+ T cells target those regions of JCPyV which are more conserved [73]. Evidence suggests that the availability of JCPyV-specific cytotoxic T cells leads to effective recovery from PML [73,74,75,76,77]. Indeed, generating JCPyV antigen-specific cytotoxic T cells and introducing them to one PML patient via adoptive transfer in conjunction with a serotonin reuptake inhibitor and another antiviral led to an improvement in symptoms and clearance of JCPyV from cerebrospinal fluid [78].

There is currently no specific antiviral drug against JCPyV but use of antiretroviral drugs in HIV patients and early detection and monitoring have played a significant role in reducing PML mortality [30,79]. Numerous drugs have been tested for efficacy against JCPyV, but none have been consistently successful thus far [80]. One candidate called Retro-2.1 (a retrograde transport inhibitor) was able to reduce in vivo murine polyomavirus infection in mice and has in the past inhibited JCPyV in vitro, but this method has yet to be translated to human treatments [81,82]. Instead, what is currently considered the best option for slowing the effects of PML is restoration of the immune system, although as stated above this increases the risk of IRIS.

Because few options are available beyond immune reconstitution, treatment of PML associated with HIV or lymphoproliferative disease—for which immune restoration may not be immediately feasible—is challenging, to say the least. One strategy involves boosting specific antiviral responses temporarily until the immune system is restored, such as through checkpoint inhibitors [83,84]. One target with generally favorable results has been the cell surface receptor programmed cell death 1 (PD-1) and associated ligand PD-L1 which function to promote CD8+ T cell exhaustion. By blocking receptor/ligand binding using monoclonal antibodies pembrolizumab or nivolumab, several patients responded with improvements—although fewer than two dozen patients in total have been treated with PD-1 inhibitors thus far [84,85,86,87,88,89,90,91,92]. One disadvantage to some types of checkpoint therapy is worsening of condition due to resultant inflammation; while this does not seem to be the case with HIV- or lymphoproliferative-associated PML, vigilance is important [84].

T cell immunotherapy has been a highly effective treatment for many cancers and offers a unique opportunity for treating viral infection as well. A creative approach to PML treatment takes advantage of the fact that JCPyV and BKPyV share about 75% of their genomic sequence—as a result, reactivity to one could potentially facilitate reactivity to the other [93,94]. While T cell immunotherapy can be expensive, a new therapeutic concept—virus-specific T cells sourced from donors and cryopreserved in a bank—partially offsets this cost and is faster than custom T cell design [95]. Using bank-sourced cytotoxic T cells specific for BKPyV in patients with PML, researchers were able to see cross-reactivity against JCPyV such that two of the three patients treated experienced clinical improvement, although evidence of IRIS (potentially linked to the treatment) was also seen in two of the three patients [96]. The use of BKPyV specific T cells sourced from outside the patient without significant adverse effects would be significant considering the high prevalence of BKPyV and its prior use in T cell immunotherapy, though the data available is currently too limited to draw concrete conclusions [95]. With further refinement use of off-the-shelf T cells may provide a potentially accessible option for suppression of PML [96,97].

### 2.4. Other JCPyV-Associated Diseases

PML is not the only disease caused by JCPyV. The virus can in rare cases lead to JCPyV granule cell neuronopathy (GCN), JCPyV encephalitis, JCPyV meningitis and JCPyV-associated nephropathy [98]. GCN is similar to PML in that it is caused by JCPyV infection of the brain, although in GCN the virus infects the granule cells of the cerebellum instead of oligodendrocytes or astrocytes [99]. Because the infections both occur in the CNS, the comorbidity of PML and GCN is likely high [100]. JC virus encephalitis and meningitis are very rare but seem to be related to the immunocompromised state of the individual and in the kidney, JCPyV-associated nephropathy is also rare, with milder symptoms than other forms of viral nephropathy [101,102,103].

## 3. Mechanisms of Disease

### 3.1. Initial Infection

**Figure 1:**
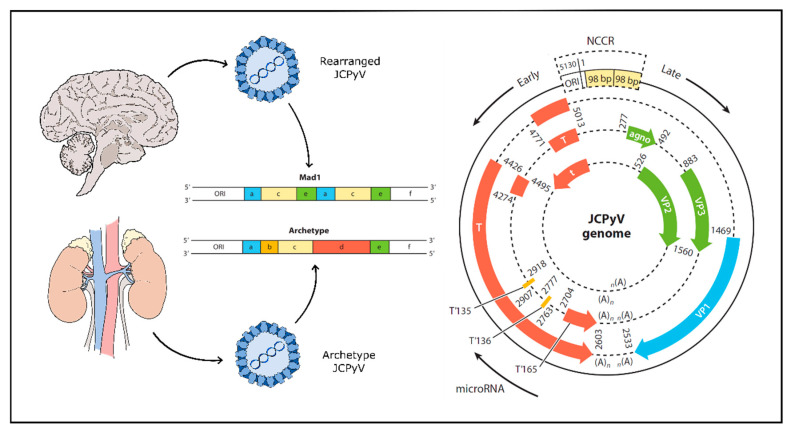
JCPyV genome undergoes rearrangement in progressive multifocal leukoencephalopathy (PML). Left panel: Archetype JCPyV develops a persistent infection in the kidneys and is generally asymptomatic. PML-type JCPyV is a rearranged form with one or more deletions or duplications of genomic block sequence in the non-coding control region (NCCR) (shown above in expanded form as a deletion of blocks b and d and a duplication of blocks a, c and e) and is found in the brain, cerebrospinal fluid (CSF) and blood. Right panel: Early genes include large T and small t antigens and are transcribed first. Late genes include structural proteins and additional regulatory proteins. Early and late genes are separated by the NCCR and are transcribed in opposite directions. ORI—origin of replication; NCCR—non-coding control region; T—T antigen and splice variants. Agno: JCPyV agnoprotein. Adapted from [30] and [126].

Initial asymptomatic infection with JCPyV likely occurs via a fecal-oral route; the virus is routinely shed in the urine and possibly in the stool [104,105,106,107,108,109]. Initial infection via tonsillar tissue has also been described, though is relatively rare [110,111,112,113,114]. Some recent research suggests the possibility of mother-to-child transmission [115]. The transmitted form of JCPyV found in urine and in kidney tissues is not generally pathogenic, although it is the most common strain of JCPyV. This form of the virus, termed the archetype strain, establishes an asymptomatic state of persistent infection for life [116]. The pathogenic form of the virus, termed the PML-type, arises following extensive genomic rearrangement of the viruses non-coding control region (NCCR) via duplications or deletions—particularly deletions within the ‘d’ block of the NCCR [117,118,119] (Figure 1). These rearrangements lead to a virus capable of high level replication in glial cells and are the only viruses found in brain parenchyma and cerebral spinal fluid of patients with active disease [20,120,121,122,123,124,125,126]. A screen of JCPyV isolated from the neural tissue of 29 individuals with PML showed the samples to be highly unique within the NCCR indicating that viral rearrangement and subsequent pathogenicity occurred after primary infection, not before [127]. These genomic rearrangements are thought to be driven by profound immunosuppression (e.g., AIDS) or by an absence of normal immune surveillance of CNS tissue (e.g., autoimmune diseases treated with immunomodulatory drugs) [119].

### 3.2. Neuroinvasion

The mode of viral trafficking from the periphery to the nervous system is not well understood, but several potential routes have been suggested. One hypothesis suggests that the virus enters the bone marrow and undergoes genomic rearrangement in cells expressing high levels of recombination enzymes, such as B cells, which then leads to viral infection of the CNS [71,128]. This would partially explain the restriction of JCPyV infection to glial cells in the brain, as many transcription factors important for infection in glial cells are also found in B cells [129,130,131]. In addition, recent work provides strong evidence that JCPyV may enter neural tissue more directly by way of the choroid plexus. Typically, the blood–brain barrier and blood–cerebrospinal fluid barrier work together to prevent everything except essential molecules from passing into neural tissue. The choroid plexus is the complex membranous structure forming the protective barrier between peripheral blood and cerebrospinal fluid (CSF) and is made up of a core (called the stroma) and a thin layer of epithelial cells [132]. Interestingly, primary choroid plexus epithelial cells express all the receptors necessary for JCPyV infection, show susceptibility to JCPyV infection in vitro and are infected in patient tissue samples in vivo, all of which suggest cells of the choroid plexus support the JCPyV viral life cycle [133,134,135]. As JCPyV enters the CNS, the virus may also directly spread from cell-to-cell along myelin sheaths [136]. An alternative hypothesis is that a small minority of individuals harbor persistent virus in brain parenchyma or choroid plexus, and it is this virus that gives rise to disease following loss of normal immune surveillance of the CNS. While one research group did report finding JCPyV genomes in the CNS of older patients without predisposing conditions, as of yet there is little data supporting this alternative hypothesis [137].

### 3.3. Animal Models

Complete animal models of JCPyV are not available which makes identifying the biologic route of infection of the virus challenging. The difficulty in generating a JCPyV animal model lies in the need for host-specific factors for viral replication; without these human-specific replication proteins, JCPyV remains highly host-restricted and unable to sustain an infection in any animal model tested to date [138,139,140,141]. Even in human cell cultures, propagation of JCPyV is an arduous task—accordingly, animal models present an even larger barrier to overcome [142]. One promising mouse model was developed by engrafting human glial progenitor cells into immunodeficient neonatal mice [141]. While this humanized chimeric model presents a significant step forward, it remains limited in providing a full understanding of the viral life cycle of JCPyV.

## 4. Hijacking the Host Genome for Replication

### 4.1. Viral Genome

JCPyV is a non-enveloped virus with a circular, dsDNA genome typically 5130 bp in size, enclosed by an icosahedral capsid [93]. The viral capsid is composed of 72 pentamers of viral protein 1 (VP1), each bound to either a single viral protein 2 (VP2) or viral protein 3 (VP3) [143]. The viral genome is well characterized and is composed of an early gene segment and a late gene segment (Figure 1). Additionally, separating the two is a non-coding control region (NCCR) containing the origin of replication as well as promoter and enhancer elements [126,144]. The early genes are transcribed first on one strand and produce large T antigen, small t antigen and three T antigen splice variants. On the complementary strand, the late genes are transcribed into three structural proteins (VP1, VP2 and VP3) as well as an agnoprotein and two micro-RNAs (miRNAs) acting to downregulate large T antigen in a negative feedback loop [145,146]. The NCCR defines the character of the virus, as rearrangement of this region is associated with increased viral replication and subsequent disease. The transmitted form of JCPyV is known as ‘archetype’ JCPyV, which refers to a specific sequence in the NCCR and which is found in the kidney, but rarely in the brain of PML patients. In contrast, a “rearranged” NCCR carries one of several altered genetic sequences that confer a growth advantage for the virus in human glia making it more likely to cause PML [116,121,122,123,126] (Figure 1). Like all viruses, JCPyV requires host cell machinery to replicate. JCPyV does this primarily through the large T antigen protein, which once transcribed interacts with a series of cellular proteins to induce viral replication and assembly [126,147].

### 4.2. Viral Entry

**Figure 2:**
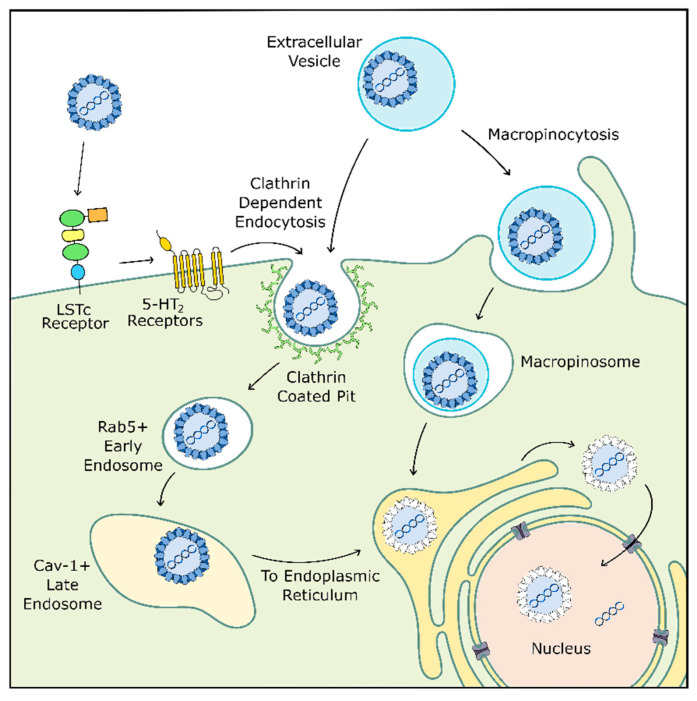
JCPyV cellular entry may occur through multiple pathways. JCPyV initially binds to LSTc receptors before transient interaction with 5-HT_2_ receptors which facilitate internalization through clathrin-mediated endocytosis. JCPyV then switches from a Rab5+ early endosome to a caveolin-1+ late endosome before entering the endoplasmic reticulum (ER) where the virus is uncoated. JCPyV exits the ER into the cytosol before entering the nucleus for replication. Alternatively, JCPyV may bind to the cell within an extracellular vesicle. Here, the vesicle is internalized through micropinocytosis or clathrin-mediated endocytosis before trafficking to the ER. Adapted from [30] and [126].

Before the virus can coopt host replication machinery, it must enter the cell (Figure 2). JCPyV initially binds to the target cell via α2,6-linked glycan lactoseries tetrasaccharide c (LSTc), a receptor motif which has both a necessary α2,6-linked sialic acid and a distinctive L-shape [148,149]. At this early stage, JCPyV may also interact with adipocyte plasma membrane associated protein (APMAP), a surface protein found in glial cells and throughout the body which appears to be important for productive viral infection [150]. JCPyV also transiently interacts with 5-hydroxytryptamine 2 receptors (5-HT2R) before clathrin-mediated endocytosis into the cell and subsequent trafficking into Rab-5 positive early endosomes [151,152,153,154]. Next, although one may expect JCPyV to traffic through Rab7-positive late endosomes, the virus instead transitions to caveolin-1 positive late endosomes [155]. This shift in endocytic pathway could be caused by several mechanisms, including those involving 5-HT2Rs, as 5-HT2Rs are also known to be recycled through clathrin-mediated endocytosis [156].

After cellular entry, JCPyV likely uses microtubules or microfilaments to traffic through the cell to the endoplasmic reticulum (ER), where the viral capsid proceeds to degrade due to resident proteins in the ER. The virus then uses the ER-associated degradation pathway to shuttle from the ER to the cytoplasm before entering the nucleus. Here, JCPyV finally releases its internal DNA and begins replication [157,158,159].

### 4.3. Viral Replication

The similarities between JCPyV and other polyomaviruses like BKPyV and simian virus 40 (SV40) again becomes useful when examining the details of the viral replication process. The replication mechanisms of SV40 have been extensively studied and likely overlap significantly with those of JCPyV [160]. Once viral DNA is present in the nucleus, regulatory proteins stall the cell cycle in S phase, providing access to cellular replication machinery and promoting an optimal environment for high-yield viral production. Like other polyomaviruses, JCPyV depends heavily on the action of large T antigen [161]. In SV40, large T antigen is made up of two phosphorylation domains and several regulatory domains, including the N-terminal DnaJ and LxCxE domains which work together to inhibit cell cycle progression and aid in DNA replication through interaction with the tumor suppressor retinoblastoma-associated protein (RB) [147,162,163]. Other regulatory regions include a threonine–proline–proline–lysine (TPPK) domain, a nuclear localization signal (NLS), an origin binding domain (OBD) and a helicase domain [164,165]. Large T antigen interacts with viral DNA as a pair of hexamers, unwinding DNA to prepare for DNA polymerase binding as well as effecting functions through interaction with proteins like p53, which contributes to cell cycle arrest [166,167,168,169,170]. Although there is significant sequence similarity between SV40 and JCPyV, there are distinct differences in biologic phenotype between the two—due in part to viral transcriptional control—so using SV40 to model viral replication mechanisms of JCPyV is helpful, but not absolute [138,171].

It is becoming clear that the smaller proteins involved in viral replication are far from insignificant. SV40 small t antigen consists of an N-terminal DnaJ domain which overlaps with the large T antigen DnaJ domain sequence and binds to the protein phosphatase PP2A to stimulate viral DNA replication. Though important, small t antigen is not necessary for SV40 replication to occur [172,173]. In JCPyV, small t antigen also interacts with PP2A and in addition to the DnaJ domain it carries two unexpected LxCxE domains at the C-terminal which likely have similar function to those found in SV40 large T antigen [174]. When JCPyV small t antigen is removed, replication efficiency originally is comparable to how SV40 would behave, but later was significantly inhibited, suggesting JCPyV may depend more heavily on its version of the small t antigen [174,175]. The three T’ spliced variants found in the JCPyV early genome additionally contribute to replication activity [176]. Together, these proteins function to arrest cell cycle progression and prepare the cell for viral replication.

New evidence suggests that JCPyV may utilize structures called “viral assembly factories” in the nucleus, which are regions where both viral DNA replication and viral packaging occur [177,178]. In BKPyV and SV40, active viral synthesis was linked to structures called promyelocytic nuclear bodies (PML-NBs) which are associated with transcriptional regulation and the DNA damage response in the cell [179]. A study of murine polyomavirus found similar results and was able to show that large T antigen, viral DNA and a host DNA repair protein could be found in these PML-NBs [177]. In the same study, tubular structures in the nucleus of infected cells were identified and postulated to be part of the viral assembly factory. Encouragingly, JCPyV seems to operate under similar mechanisms. JCPyV not only recruits cellular DNA damage-response proteins to sites of viral replication; it also replicates its DNA within similar tubular structures composed of VP1 [178,180,181]. These VP1 structures are similar to those identified in the brain tissue of individuals with PML in earlier histological studies, suggesting they are a factor in pathologic JCPyV replication [178,182,183].

Together, these data give clarity to the process of JCPyV virion assembly in the nucleus. After reaching the nucleus, JCPyV expresses its early genes which work in concert to halt the cell in S phase and recruit cell DNA damage-response proteins to a localized area adjacent to cell PML-NBs termed the viral assembly factory. As JCPyV late genes are transcribed, VP1 tubules begin to form within the nucleus, and JCPyV agnoprotein is produced to assist in DNA replication and promote viral release through mechanisms similar to other viroporins [184,185,186]. Agnoprotein may also play a role in modulating host immune response, as its release from infected cells leads to a reduction of activating cytokine from neighboring cells as well as potentially inhibiting the response of local immune cells, which could explain the lack of generalized inflammation in the CNS of patients with PML [187].

### 4.4. Serotonin Receptors and JCPyV

Serotonin type 2 receptors (5-HT2Rs) have the potential to play a role in multiple stages of JCPyV infection, from immediately after LSTc binding (viral attachment) to later trafficking within endosomes. 5-HT2Rs are one of seven 5-HT receptor families, each with a variety of lettered subtypes (2A, B and C, for example). There are a total of 14 distinct 5-HT receptors. All 5-HT receptors are G-protein coupled receptors (GPCRs) excepting the 5-HT3A receptor, which is an ion-gated channel [154,156,188]. GPCRs are known for having widespread effects in the cell and within body systems and 5-HT2Rs in particular are expressed in a variety of cell types, including astrocytes, oligodendrocytes, neurons, kidney epithelial cells and choroid plexus epithelial cells [133]. The presence of 5-HT2Rs on so many cell types does not indicate viral permissivity; it is thought that 5-HT2Rs are used only after JCPyV has already bound to an LSTc motif or has otherwise bound itself to the cell surface. Due to the multitude of points at which 5-HT2Rs may play a role in JCPyV infection, it is possible that targeting viral spread through inhibition of these receptors may be one route to therapy or treatment for PML, though additional research is necessary to determine the effectiveness of this approach [189].

JCPyV uses the three subtypes of 5-HT2R (5-HT2AR, 5-HT2BR and 5-HT2CR) to infect cells and these three receptors may fulfill complementary roles [153,154,189,190]. CRISPR-Cas9 knockout mutations of the receptor proteins showed that removal of the second intracellular loop of the receptor inhibits infection, potentially through disruption of receptor internalization through a β-arrestin pathway [190,191]. Because it is unclear what type of interaction JCPyV has with 5-HT2Rs, it is likely that understanding the way 5-HT2Rs interact with each other will shed light on the process of JCPyV cellular entry.

### 4.5. Extracellular Vesicles and JCPyV

A central paradox regarding JCPyV infection is that although the LSTc receptor was shown to be necessary for viral binding to the cell surface, glial cells in the brain (oligodendrocytes and astrocytes) do not express LSTc receptors and do not bind virus [133,134]. In addition, rearranged variants of JCPyV from the CNS of PML patients were found to contain mutations in the LSTc-binding pocket of VP1, which renders them incapable of binding to the sialic acid containing attachment receptor LSTc [122,123,149,192]. These mutations are thought to allow the virus to evade the host immune response, but this would not be beneficial for JCPyV if it prevented viral propagation altogether [125]. Some research suggests that these mutants may use non-sialylated glycosaminoglycans (GAG) as an alternative entry receptor, although it is unclear which specific part of the GAG structure is utilized [193].

An alternative hypothesis involves the virus’ ability to exploit receptor-independent modes of infection. In this case, maintaining receptor function as such would not be necessary for the virus, and mutations accumulated in the viral receptor binding sites would not affect viral propagation [134,194]. Extracellular vesicles (EVs) are known to play important roles in cell communication and more recently for their role in transmitting viruses like JCPyV [194,195,196,197,198]. EVs are secreted vesicles that may travel between cell types in an organ or be excreted. These vesicles can contain anything from protein to RNA to lipids and assist greatly in modulating homeostasis [198]. Several viruses, including HIV-1, HSV-1, HepA virus and norovirus, use EVs as a “transportation highway”, delivering infectious viral particles in a highly efficient manner between cells [199,200,201,202,203,204,205].

**Figure 3:**
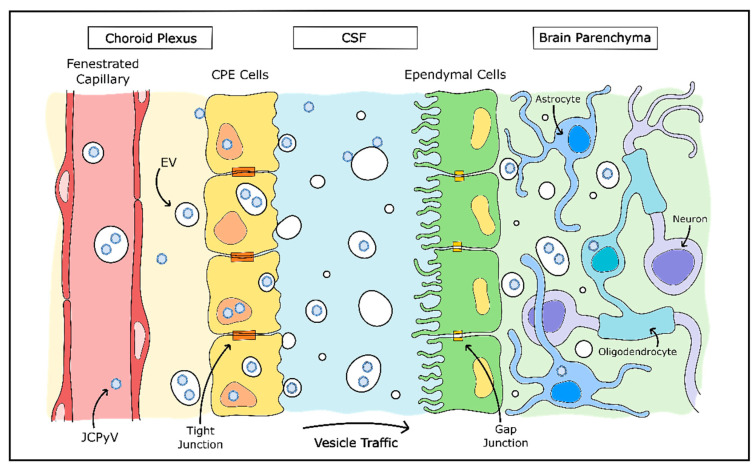
Model of JCPyV CNS entry by choroid plexus. Evidence suggests JCPyV may utilize extracellular vesicles (EVs) in entry of the CNS. A model proposed by O’Hara et al. theorizes JCPyV may pass into the stroma from the bloodstream as either free virions or enclosed in capsules. JCPyV is then able to infect choroid plexus epithelial (CPE) cells which package and release the virions in EVs into the CSF. JCPyV-containing EVs then enter the brain parenchyma and infect glial cells without necessitating the use of the LSTc cellular entry receptor. Adapted from [194].

JCPyV is among those viruses which make use of EVs and may explain much of the mystery around JCPyV infection of cells in the CNS (Figure 3). JCPyV was found to associate both with the exterior of EVs and also packaged inside EVs, and these virus-associated EVs were highly infectious to cultured glial cells in a receptor-independent manner. In addition, viral packaging within the EVs protected JCPyV from neutralizing antisera [205,206]. EVs containing archetype-form JCPyV were recently isolated from human plasma, which also lends credence to the importance of EVs for JCPyV infection [197]. The presence of EV-mediated viral infection is a promising explanation for how oligodendrocytes and astrocytes are infected by JCPyV without the presence of attachment receptors and may even explain how the virus enters the CNS originally.

As discussed earlier, the attachment receptor for JCPyV (LSTc) is not found on oligodendrocytes or astrocytes, but is found abundantly in choroid plexus epithelial cells, as well as in microglia and brain microvascular endothelial cells [133]. Choroid plexus epithelial cells were found to be susceptible to JCPyV infection in vitro, are also permissible to in vivo infection and may mediate JCPyV transfer across the blood–cerebrospinal fluid barrier [134,207]. In addition, EVs released by JCPyV-infected immortalized choroid plexus epithelial cells contained complete JCPyV particles. These EVs were consistently capable of infecting in vitro glial cells in a receptor-independent manner—instead, entering the cell via clathrin-mediated endocytosis and macropinocytosis [194]. The hypothesis that JCPyV infection of the CNS is largely mediated by EVs is supported by other findings that choroid plexus epithelial cells release EVs into the cerebrospinal fluid, particularly in inflammatory environments and have the capability to transmit the contents of these released EVs to astrocytes [208]. Although this method of vesicular communication may normally be used to convey information to brain tissue in times of peripheral stress, it is possible that JCPyV uses this same system to enter the CNS without detection from the host immune system.

Application of drugs that inhibit neutral sphingomyelinase (GW4869 and cambinol) reduce the release of EVs from choroid plexus epithelial cells in vitro and more generally reduced EVs in the brain and serum in vivo, but this may not be applicable in reducing pathogenic viral spread, as EVs have also been shown to have a protective effect for neighboring cells in viral infection [203,208,209,210]. Further examination of the role EVs play in mediating JCPyV spread in PML may lead to potential clinical interventions to reduce the likelihood of developing PML for those individuals at risk.

## 5. Conclusions

Soon, viral-like particles (VLPs) may be used to develop a vaccine against pathogenic JCPyV, eliminating the risk of developing PML altogether. Examination of sera from healthy and PML-affected individuals suggest that PML may arise when humoral immunity is unable to identify JCPyV pathogenic variants; subsequent generation of a vaccine using VLPs and administration in mice and one human patient led to heightened antibody titers against the strain of pathogenic JCPyV [20,125]. Specific VLPs used as a vaccine before the risk of development of PML may therefore have a protective effect against the disease, however more work needs to be done in developing the antibodies to be used for preventative treatment [211]. Additionally, modifying JCPyV into another type of VLP takes advantage of the limited infectious range of the virus and opens the door to its use as a vector in gene therapy [212]. Excitingly, this method was used more recently with success in the treatment of glioblastoma multiforme and even prostate cancer in mice [213,214]. As JCPyV is better understood as a virus and as a vector, the scientific community may see its transition from being the causative agent in disease to a contributing factor in treatment.

JCPyV often uses LSTc as an attachment receptor before associating with 5-HT2 receptors during cellular entry, but whether LSTc is on a lipid, a protein or both is not known and alternative receptors or EVs may be used to assist viral spread. Understanding these alternative infection pathways in the context of PML may hold the key to targeting and preventing pathogenic JCPyV spread.

Although in most cases JCPyV infection is asymptomatic, when it does become pathogenic, the virus can be extremely deadly. It is critical we continue striving to understand the biology of JCPyV so that the risk of developing PML and other JCPyV-associated diseases can be effectively reduced or eliminated. Because pathogenic JCPyV takes advantage of an immunosuppressed state in an individual, determining a method to reduce the likelihood of developing PML will both reduce the risk of death from HIV-associated or hematological disease and make the use of immunosuppressive drugs to treat diseases like multiple sclerosis far safer.

## References

[1] Gross L. (1953). A filterable agent, recovered from Ak leukemic extracts, causing salivary gland carcinomas in C3H mice. Proc. Soc. Exp. Biol. Med..

[2] Stewart S.E., Eddy B.E., Gochenour A.M., Borgese N.G., Grubbs G.E. (1953). Leukemia in mice produced by a filterable agent present in AKR leukemic tissues with notes on a sarcoma produced by the same agent. Anat. Rec..

[3] Sweet B.H., Hilleman M.R. (1960). The Vacuolating Virus, S.V.40. Proc. Soc. Exp. Biol. Med..

[4] Lehn H., Müller H. (1986). Cloning and characterization of budgerigar fledgling disease virus, an avian polyomavirus. Virology.

[5] Johne R., Buck C.B., Allander T., Atwood W.J., Garcea R.L., Imperiale M.J., Major E.O., Ramqvist T., Norkin L.C. (2011). Taxonomical developments in the family *Polyomaviridae*. Arch. Virol..

[6] Pinto M., Dobson S. (2014). BK and JC virus: A review. J. Infect..

[7] Killham L. (1952). Isolation in suckling mice of a virus from C3H mice harboring Bittner milk agent. Science.

[8] Gardner S.D., Field A.M., Coleman D.V., Hulme B. (1971). New human papovavirus (B.K.) isolated from urine after renal transplantation. Lancet.

[9] Padgett B.L., Walker D.L., ZuRhein G.M., Eckroade R.J., Dessel B.H. (1971). Cultivation of papova-like virus from human brain with progressive multifocal leucoencephalopathy. Lancet.

[10] Allander T., Andreasson K., Gupta S., Bjerkner A., Bogdanovic G., Persson M.A., Dalianis T., Ramqvist T., Andersson B. (2007). Identification of a third human polyomavirus. J. Virol..

[11] Gaynor A.M., Nissen M.D., Whiley D.M., Mackay I.M., Lambert S.B., Wu G., Brennan D.C., Storch G.A., Sloots T.P., Wang D. (2007). Identification of a novel polyomavirus from patients with acute respiratory tract infections. PLoS Pathog..

[12] Feng H., Shuda M., Chang Y., Moore P.S. (2008). Clonal integration of a polyomavirus in human Merkel cell carcinoma. Science.

[13] Schowalter R.M., Pastrana D.V., Pumphrey K.A., Moyer A.L., Buck C.B. (2010). Merkel cell polyomavirus and two previously unknown polyomaviruses are chronically shed from human skin. Cell Host Microbe.

[14] van der Meijden E., Janssens R.W.A., Lauber C., Bavinck J.N.B., Gorbalenya A.E., Feltkamp M.C.W. (2010). Discovery of a new human polyomavirus associated with trichodysplasia spinulosa in an immunocompromized patient. PLoS Pathog..

[15] Scuda N., Hofmann J., Calvignac-Spencer S., Ruprecht K., Liman P., Kühn J., Hengel H., Ehlers B. (2011). A novel human polyomavirus closely related to the african green monkey-derived lymphotropic polyomavirus. J. Virol..

[16] Buck C.B., Phan G.Q., Raiji M.T., Murphy P.M., McDermott D.H., McBride A.A. (2012). Complete genome sequence of a tenth human polyomavirus. J. Virol..

[17] Lim E.S., Reyes A., Antonio M., Saha D., Ikumapayi U.N., Adeyemi M., Stine O.C., Skelton R., Brennan D.C., Mkakosya R.S. (2013). Discovery of STL polyomavirus, a polyomavirus of ancestral recombinant origin that encodes a unique T antigen by alternative splicing. Virology.

[18] Korup S., Rietscher J., Calvignac-Spencer S., Trusch F., Hofmann J., Moens U., Sauer I., Voigt S., Schmuck R., Ehlers B. (2013). Identification of a novel human polyomavirus in organs of the gastrointestinal tract. PLoS ONE.

[19] Mishra N., Pereira M., Rhodes R.H., An P., Pipas J.M., Jain K., Kapoor A., Briese T., Faust P.L., Lipkin W.I. (2014). Identification of a novel polyomavirus in a pancreatic transplant recipient with retinal blindness and vasculitic myopathy. J. Infect. Dis..

[20] Haley S.H., Atwood W.J. (2017). Progressive Multifocal Leukoencephalopathy: Endemic Viruses and Lethal Brain Disease. Annu. Rev. Virol..

[21] Padgett B.L., Walker D.L. (1973). Prevalence of antibodies in human sera against JC virus, an isolate from a case of progressive multifocal leukoencephalopathy. J. Infect. Dis..

[22] Chang H., Wang M., Tsai R.T., Lin H.S., Huan J.S., Wang W.C., Chang D. (2002). High incidence of JC virurea in JC-seropositive older individuals. J. Neurovirol..

[23] Kean J.M., Rao S., Wang M., Garcea R.L. (2009). Seroepidemiology of human polyomaviruses. PLoS Pathog..

[24] Knowles W.A., Pipkin P., Andrews N., Vyse A., Minor P., Brown D.W., Miller E. (2003). Population-based study of antibody to the human polyomaviruses BKV and JCV and the simian polyomavirus SV40. J. Med. Virol..

[25] Egli A., Infanti L., Dumoulin A., Buser A., Samaridis J., Stebler C., Gosert R., Hirsch H.H. (2009). Prevalence of Polyomavirus BK and JC Infection and Replication in 400 Healthy Blood Donors. J. Infect. Dis..

[26] Aksamit A.J., Major E.O., Ghatak N.R., Sidhu G.S., Parisi J.E., Guccion J.G. (1987). Diagnosis of progressive multifocal leukoencephalopathy by brain biopsy with biotin labeled DNA: DNA in situ hybridization. J. Neuropathol. Exp. Neurol..

[27] Berger J.R., Major E.O. (1999). Progressive Multifocal Leukoencephalopathy. Semin. Neurol..

[28] Åström K.E., Mancall E.L., Richardson E.P. (1958). Progressive multifocal leukoencephalopathy; a hitherto unrecognized complication of chronic lymphatic leukaemia and Hodgkin’s disease. Brain.

[29] Brooks B.R., Walker D.L. (1984). Progressive Multifocal Leukoencephalopathy. Neurol. Clin..

[30] Ferenczy M.W., Marshall L.J., Nelson C.D., Atwood W.J., Nath A.J., Khalili K., Major E.O. (2012). Molecular biology, epidemiology, and pathogenesis of progressive multifocal leukoencephalopathy, the JC virus-induced demyelinating disease of the human brain. Clin. Microbiol. Rev..

[31] Sikkema T., Schuiling W.J., Hoogendoorn M. (2013). Progressive multifocal leukoencephalopathy during treatment with rituximab and CHOP chemotherapy in a patient with a diffuse large B-cell lymphoma. BMJ Case Rep..

[32] Berger J.R., Askamit A.J., Clifford D.B., Davis L., Koralnik I.J., Sejvar J.J., Bartt R., Major E.O., Nath A. (2013). PML diagnostic criteria: Consensus statement from the AAN Neuroinfectious Disease Section. Neurology.

[33] Cavanagh J.B., Greenbaum D., Marshall A.H., Rubinstein L.J. (1959). Cerebral demyelination associated with disorders of the reticuloendothelial system. Lancet.

[34] Richardson E.P. (1961). Progressive Multifocal Leukoencephalopathy. N. Engl. J. Med..

[35] Holman R.C., Torok T.J., Belay E.D., Janssen R.S., Schonberger L.B. (1998). Progressive multifocal leukoencephalopathy in the United States, 1979–1994: Increased mortality associated with HIV infection. Neuroepidemiology.

[36] Berger J.R., Kaszovitz B., Post M.J.D., Dickinson G. (1987). Progressive Multifocal Leukoencephalopathy Associated with Human Immunodeficiency Virus Infection: A Review of the Literature with a Report of Sixteen Cases. Ann. Intern. Med..

[37] Rhodes R.H., Ward J.M., Walker D.L., Ross A.A. (1988). Progressive multifocal leukoencephalopathy and retroviral encephalitis in acquired immunodeficiency syndrome. Arch. Path..

[38] Aksamit A.J., Gendelman H.E., Orenstein J.M., Pezeshkpour G.H. (1990). AIDS-associated progressive multifocal leukoencephalopathy (PML): Comparison to non-AIDS PML with in-situ hybridization and immunohistochemistry. Neurology.

[39] Sacktor N. (2002). The epidemiology of human immunodeficiency virus–associated neurological disease in the era of highly active antiretroviral therapy. J. Neurovirol..

[40] Major E.O. (2010). Progressive multifocal leukoencephalopathy in patients on immunomodulatory therapies. Annu. Rev. Med..

[41] Christensen K.L., Holman R.C., Hammett T.A., Belay E.D., Schonberger L.B. (2010). Progressive multifocal leukoencephalopathy deaths in the USA, 1979–2005. Neuroepidemiology.

[42] Berger J.R., Levy R.M., Flomenhoft D., Dobbs M. (1998). Predictive Factors for Prolonged Survival in Acquired Immunodeficiency Syndrome—Associated Progressive Multifocal Leukoencephalopathy. Ann. Neurol..

[43] Berenguer J., Miralles P., Arrizabalaga J., Ribera E., Dronda F., Baraia-Etxaburu J., Domingo P., Márquez M., Rodriguez-Arrondo F.J., Laguna F. (2003). Clinical Course and Prognostic Factors of Progressive Multifocal Leukoencephalopathy in Patients Treated with Highly Active Antiretroviral Therapy. Clin. Infect. Dis..

[44] Cinque P., Bossolasco S., Brambilla A.M., Boschini A., Mussini C., Pierotti C., Campi A., Casari S., Bertelli D., Mena M. (2003). The effect of highly active antiretroviral therapy induced immune reconstitution on development and outcome of progressive multifocal leukoencephalopathy: Study of 43 cases with review of the literature. J. Neurovirol..

[45] Gasnault J., Costagliola D., Hendel-Chavez H., Dulioust A., Pakianather S., Mazet A.A., de Goer de Herve M.G., Lancar R., Lascaux A.S., Porte L. (2011). Improved survival of HIV-1-infected patients with progressive multifocal leukoencephalopathy receiving early 5-drug combination antiretroviral therapy. PLoS ONE.

[46] Kleinschmidt-DeMasters B.K., Tyler K.L. (2005). Progressive multifocal leukoencephalopathy complicating treatment with natalizumab and interferon beta-1a for multiple sclerosis. N. Engl. J. Med..

[47] Kartau M., Verkkoniemi-Ahola A., Paetau A., Palomäki M., Janes R., Ristola M., Lappalainen M., Anttila V.J. (2019). The Incidence and Predisposing Factors of John Cunningham Virus-Induced Progressive Multifocal Leukoencephalopathy in Southern Finland: A Population-Based Study. Open Forum Infect. Dis..

[48] Steiner I., Berger J.R. (2012). Update on progressive multifocal leukoencephalopathy. Curr. Neurol. Neurosci..

[49] Post M.J., Thurnher M.M., Clifford D.B., Nath A., Gonzalez R.G., Gupta R.K., Post K.K. (2013). CNS-immune reconstitution inflammatory syndrome in the setting of HIV infection, part 1: Overview and discussion of progressive multifocal leukoencephalopathy-immune reconstitution inflammatory syndrome and cryptococcal-immune reconstitution inflammatory syndrome. Am. J. Neuroradiol..

[50] Clifford D.B. (2015). Neurological immune reconstitution inflammatory response: Riding the tide of immune recovery. Curr. Opin. Neurol..

[51] Berger J.R. (2006). Natalizumab and progressive multifocal leucoencephalopathy. Ann. Rheum. Dis..

[52] Williamson E.M.L., Berger J.R. (2015). Infection risk in patients on multiple sclerosis therapeutics. CNS Drugs.

[53] Williamson E.M.L., Berger J.R. (2017). Diagnosis and Treatment of Progressive Multifocal Leukoencephalopathy Associated with Multiple Sclerosis Therapies. Neurotherapeutics.

[54] Langer-Gould A., Atlas S.W., Green A.J., Bollen A.W., Pelletier D. (2005). Progressive Multifocal Leukoencephalopathy in a Patient Treated with Natalizumab. N. Engl. J. Med..

[55] Van Assche G., Van Ranst M., Sciot R., Dubois B., Vermeire S., Noman M., Verbeeck J., Geboes K., Robberecht W., Rutgeerts P. (2005). Progressive Multifocal Leukoencephalopathy after Natalizumab Therapy for Crohn’s Disease. N. Engl. J. Med..

[56] Kothary N., Diak I.L., Brinker A., Bezabeh S., Avigan M., Dal Pan G. (2011). Progressive multifocal leukoencephalopathy associated with efalizumab use in psoriasis patients. J. Am. Acad. Dermatol..

[57] Bloomgren G., Richman S., Hotermans C., Subramanyam M., Goelz S., Natarajan A., Lee S., Plavina T., Scanlon J.V., Sandrock A. (2012). Risk of Natalizumab-Associated Progressive Multifocal Leukoencephalopathy. N. Engl. J. Med..

[58] Rosas M.J., Simões-Ribeiro F., An S.F., Sousa N. (1999). Progressive multifocal leukoencephalopathy: Unusual MRI findings and prolonged survival in a pregnant woman. Neurology.

[59] Gheuens S., Pierone G., Peeters P., Koralnik I.J. (2010). Progressive multifocal leukoencephalopathy in individuals with minimal or occult immunosuppression. J. Neurol. Neurosurg. Psychiatry.

[60] Kartau M., Sipilä J.O.T., Auvinen E., Palomäki M., Verkkoniemi-Ahola A. (2019). Progressive Multifocal Leukoencephalopathy: Current Insights. Degener. Neurol. Neuromuscul. Dis..

[61] Miskin D.P., Herman S.T., Ngo L.H., Koralnik I.J. (2016). Predictors and characteristics of seizures in survivors of progressive multifocal leukoencephalopathy. J. Neurovirol..

[62] Lindå H., von Heijne A. (2013). Presymptomatic diagnosis with MRI and adequate treatment ameliorate the outcome after natalizumab-associated progressive multifocal leukoencephalopathy. Front. Neurol..

[63] Cinque P., Koralnik I.J., Gerevini S., Miro J.M., Price R.W. (2009). Progressive multifocal leukoencephalopathy in HIV-1 infection. Lancet Infect. Dis..

[64] Wijburg M.T., Witte B.I., Vennegoor A., Roosendaal S.D., Sanchez E., Liu Y., Jarnalo C.O.M., Uitdehaag B.M.J., Barkhof F., Killestein J. (2016). MRI criteria differentiating asymptomatic PML from new MS lesions during natalizumab pharmacovigilance. J. Neurol. Neurosurg. Psychiatry.

[65] Wattjes M.P., Wijburg M.T., van Eijk J., Frequin S., Uitdehaag B.M.J., Barkhof F., Warnke C., Killstein J., Dutch-Belgian Natalizumab-Associated PML Study Group (2018). Inflammatory natalizumab-associated PML: Baseline characteristics, lesion evolution and relation with PML-IRIS. J. Neurol. Neurosurg. Psychiatry.

[66] Anand P., Hotan G.C., Vogel A., Venna N., Mateen F.J. (2019). Progressive multifocal leukoencephalopathy: A 25-year retrospective cohort study. Neurol. Neuroimmunol. Neuroinflamm..

[67] Khanna N., Elzi L., Mueller N.J., Garzoni C., Cavassini M., Fux C.A., Vernazza P., Bernasconi E., Battegay M., Hirsch H.H. (2009). Incidence and outcome of progressive multifocal leukoencephalopathy over 20 years of the Swiss HIV Cohort Study. Clin. Infect. Dis..

[68] Weber F., Goldmann C., Krämer M., Kaup F.J., Pickhardt M., Young P., Petry H., Weber T., Lüke W. (2001). Cellular and Humoral Immune Response in Progressive Multifocal Leukoencephalopathy. Ann. Neurol..

[69] Koralnik I.J., Du Pasquier R.A., Kuroda M.J., Schmitz J.E., Dang X., Zheng Y., Lifton M., Letvin N.L. (2002). Association of prolonged survival in HLA-A2 + progressive multifocal leukoencephalopathy patients with a CTL response specific for a commonly recognized JC virus epitope. J. Immunol..

[70] Engsig F.N., Hansen A.E., Omland L.H., Kronborg G., Gerstoft J., Laursen A.L., Pedersen C., Mogensen C.B., Nielsen L., Obel N. (2009). Incidence, clinical presentation, and outcome of progressive multifocal leukoencephalopathy in HIV-infected patients during the highly active antiretroviral therapy era: A nationwide cohort study. J. Infect. Dis..

[71] Pavlovic D., Patel M.A., Patera A.C., Peterson I. (2018). Progressive Multifocal Leukoencephalopathy Consortium. T cell deficiencies as a common risk factor for drug associated progressive multifocal leukoencephalopathy. Immunobiology.

[72] Du Pasquier R.A., Kuroda M.J., Schmitz J.E., Zheng Y., Martin K., Peyerl F.W., Lifton M., Gorgone D., Autissier P., Letvin N.L. (2003). Low Frequency of Cytotoxic T Lymphocytes against the Novel HLA-A*0201-Restricted JC Virus Epitope VP1_p36_ in Patients with Proven or Possible Progressive Multifocal Leukoencephalopathy. J. Virol..

[73] Jelcic I., Jelcic I., Kempf C., Largey F., Planas R., Schippling S., Budka H., Sospedra M., Martin R. (2016). Mechanisms of Immune Escape in Central Nervous System Infection with Neurotropic JC Virus Variant. Ann. Neurol..

[74] Koralnik I.J., Du Pasquier R.A., Letvin N.L. (2001). JC Virus-Specific Cytotoxic T Lymphocytes in Individuals with Progressive Multifocal Leukoencephalopathy. J. Virol..

[75] Du Pasquier R.A., Clark K.W., Smith P.S., Joseph J.T., Mazullo J.M., De Girolami U., Letvin N.L., Koralnik I.J. (2001). JCV-specific cellular immune response correlates with a favorable clinical outcome in HIV-infected individuals with progressive multifocal leukoencephalopathy. J. Neurovirol..

[76] Aly L., Yousef S., Schippling S., Jelcic I., Breiden P., Matschke J., Schulz R., Bofill-Mas S., Jones L., Demina V. (2011). Central role of JC virus-specific CD4^+^ lymphocytes in progressive multi-focal leukoencephalopathy-immune reconstitution inflammatory syndrome. Brain.

[77] Gheuens S., Bord E., Kesari S., Simpson D.M., Gandhi R.T., Clifford D.B., Berger J.R., Ngo L., Koralnik I.J. (2011). Role of CD4^+^ and CD8^+^ T-Cell Responses against JC Virus in the Outcome of Patients with Progressive Multifocal Leukoencephalopathy (PML) and PML with Immune Reconstitution Inflammatory Syndrome. J. Virol..

[78] Balduzzi A., Lucchini G., Hirsch H.H., Basso S., Cioni M., Rovelli A., Zincone A., Grimaldi M., Corti P., Bonanomi S. (2011). Polyomavirus JC-targeted T-cell therapy for progressive multiple leukoencephalopathy in a hematopoietic cell transplantation recipient. Bone Marrow Transplant..

[79] Garvey L., Winston A., Walsh J., Post F., Porter K., Gazzard B., Fisher M., Leen C., Pillay D., UK Collaborative HIV Cohort (CHIC) Study Steering Committee (2011). HIV-associated central nervous system diseases in the recent combination antiretroviral therapy era. Eur. J. Neurol..

[80] Pavlovic D., Patera A.C., Nyberg F., Gerber M., Liu M. (2015). Progressive multifocal leukoencephaly: Current treatment options and future perspectives. Ther. Adv. Neurol. Disord..

[81] Nelson C.D., Carney D.W., Derdowski A., Lipovsky A., Gee G.V., O’Hara B., Williard P., DiMaio D., Sello J.K., Atwood W.J. (2013). A retrograde trafficking inhibitor of ricin and Shiga-like toxins inhibits infection of cells by human and monkey polyomaviruses. mBio.

[82] Maru S., Jin G., Desai D., Amin S., Lauver M.D., Lukacher A.E. (2017). Inhibition of Retrograde Transport Limits Polyomavirus Infection in Vivo. mSphere.

[83] Sanjo N., Nose Y., Shishido-Hara Y., Mizutani S., Sekijima Y., Aizawa H., Tanizawa T., Yokota T. (2019). A controlled inflammation and a regulatory immune system are associated with more favorable prognosis of progressive multifocal leukoencephalopathy. J. Neurol..

[84] Beck E.S., Cortese I. (2020). Checkpoint inhibitors for the treatment of JC virus-related progressive multifocal leukoencephalopathy. Curr. Opin. Virol..

[85] Audemard-Verger A., Gasnault J., Faisant M., Besse M., Martin-Silva N., Berra M., Fournier L., Boutemy J., Maigne G., de Boysson H. (2019). Sustained Response and Rationale of Programmed Cell Death-1-Targeting for Progressive Multifocal Leukoencephalopathy. Open Forum Infect. Dis..

[86] Cortese I., Muranski P., Enose-Akahata Y., Ha S., Smith B., Monaco M., Ryschkewitch C., Major E.O., Ohayon J., Schindler M.K. (2019). Pembrolizumab Treatment for Progressive Multifocal Leukoencephalopathy. N. Engl. J. Med..

[87] Hoang E., Bartlett N.L., Goyal M.S., Schmidt R.E., Clifford D.B. (2019). Progressive multifocal leukoencephalopathy treated with nivolumab. J. Neurovirol..

[88] Küpper C., Heinrich J., Kamm K., Bücklein V., Rothenfusser S., Straube A. (2019). Pembrolizumab for progressive multifocal leukoencephalopathy due to primary immunodeficiency. Neurol. Neuroimmunol. Neuroinflamm..

[89] Medrano C., Vergez F., Mengelle C., Faguer S., Kamar N., Del Bello A. (2019). Effectiveness of Immune Checkpoint Inhibitors in Transplant Recipients with Progressive Multifocal Leukoencephalopathy. Emerg. Infect. Dis..

[90] Pawlitzki M., Schneider-Hohendorf T., Rolfes L., Meuth S.G., Wiendl H., Schwab N., Grauer O.M. (2019). Ineffective treatment of PML with pembrolizumab: Exhausted memory T-cell subsets as a clue?. Neurol. Neuroimmunol. Neuroinflamm..

[91] Rauer S., Marks R., Urbach H., Warnatz K., Nath A., Holland S., Weiller C., Grimbacher B. (2019). Treatment of Progressive Multifocal Leukoencephalopathy with Pembrolizumab. N. Engl. J. Med..

[92] Walter O., Treiner E., Bonneville F., Mengelle C., Vergez F., Lerebours F., Delobel P., Liblau R., Martin-Blondel G., Immune Checkpoint Inhibitors in PML Study Group (2019). Treatment of Progressive Multifocal Leukoencephalopathy with Nivolumab. N. Engl. J. Med..

[93] Frisque R.J., Bream G.L., Cannella M.T. (1984). Human polyomavirus JC virus genome. J. Virol..

[94] Lindner J.M., Cornacchione V., Sathe A., Be C., Srinivas H., Riquet E., Leber X., Hein A., Wrobel M.B., Scharenberg M. (2019). Human Memory B Cells Harbor Diverse Cross-Neutralizing Antibodies against BK and JC Polyomaviruses. Immunity.

[95] Tzannou I., Papadopoulou A., Naik S., Leung K., Martinez C.A., Ramos C.A., Carrum G., Sasa G., Lulla P., Watanabe A. (2017). Off-the-Shelf Virus-Specific T Cells to Treat BK Virus, Human Herpesvirus 6, Cytomegalovirus, Epstein-Barr Virus, and Adenovirus Infections after Allogenic Hematopoietic Stem-Cell Transplantation. J. Clin. Oncol..

[96] Muftuoglu M., Olson A., Marin D., Ahmed S., Mulanovich V., Tummala S., Chi T.L., Ferrajoli A., Kaur I., Li L. (2018). Allogenic BK Virus-Specific T Cells for Progresive Multifocal Leukoencephalopathy. N. Engl. J. Med..

[97] Krymskaya L., Sharma M.C., Martinez J., Haq W., Huang E.C., Limaye A.P., Diamond D.J., Lacey S.F. (2005). Cross-Reactivity of T Lymphocytes Recognizing a Human Cytotoxic T-Lymphocyte Epitope within BK and JC Virus VP1 Polypeptides. J. Virol..

[98] Tan C.S., Koralnik I.J. (2010). Progressive multifocal leukoencephalopathy and other disorders caused by JC virus: Clinical features and pathogenesis. Lancet Neurol..

[99] Du Pasquier R.A., Corey S., Margolin D.H., Williams K., Pfister L.A., De Girolami U., Mac Key J.J., Wüthrich C., Joseph J.T., Koralnik I.J. (2003). Productive infection of cerebellar granule cell neurons by JC virus in an HIV+ individual. Neurology.

[100] Wüthrich C., Cheng Y.M., Joseph J.T., Kesari S., Beckwith C., Stopa E., Bell J.E., Koralnik I.J. (2009). Frequent infection of cerebellar granule cell neurons by polyomavirus JC in progressive multifocal leukoencephalopathy. J. Neuropath. Exp. Neurol..

[101] Blake K., Pillay D., Knowles W., Brown D.W.G., Griffiths P.D., Taylor B. (1992). JC virus associated meningoencephalitis in an immunocompetent girl. Arch. Dis. Child..

[102] Viallard J., Ellie E., Lazaro E., Lafon M., Pellegrin J. (2005). JC virus meningitis in a patient with systemic lupus erythematosus. Lupus.

[103] Kantarci G., Eren Z., Demirağ A., Dogan I., Çakalagaoglu F., Yilmaz G. (2011). JC virus-associated nephropathy in a renal transplant recipient and comparative analysis of previous cases. Transpl. Infect. Dis..

[104] Coleman D.V., Wolfendale M.R., Daniel R.A., Dhanjal N.K., Gardner S.D., Gibson P.E., Field A.M. (1980). A prospective study on human polyomavirus infection in pregnancy. J. Infect. Dis..

[105] Arthur R.R., Dagostin S., Shah K.V. (1989). Detection of BK virus and JC virus in urine and brain tissue by the polymerase chain reaction. J. Clin. Microbiol..

[106] Sundsfjord A., Flaegstad T., Flø R., Spein A.R., Pedersen M., Permin H., Julsrud J., Traavik T. (1994). BK and JC viruses in human immunodeficiency virus type 1-infected persons: Prevalence, excretion, viremia, and viral regulatory regions. J. Infect. Dis..

[107] Bofill-Mas S., Formiga-Cruz M., Clemente-Casares P., Calafell F., Girones R. (2001). Potential Transmission of Human Polyomaviruses through the Gastrointestinal Tract after Exposure to Virions or Viral DNA. J. Virol..

[108] Rossi A., Delbue S., Mazziotti R., Valli M., Borghi E., Manusco R., Calvo M.G., Ferrante P. (2007). Presence, quantitation and characterization of JC virus in the urine of Italian immunocompetent subjects. J. Med. Virol..

[109] Rudick R.A., O’Connor P.W., Polman C.H., Goodman A.D., Ray S.S., Griffith N.M., Jurgensen S.A., Gorelik L., Forrestal F., Sandrock A.W. (2010). Assessment of JC virus DNA in blood and urine from natalizumab-treated patients. Ann. Neurol..

[110] Monaco M.C., Atwood W.J., Gravell M., Tornatore C.S., Major E.O. (1996). JC virus infection of hematopoietic progenitor cells, primary B lymphocytes, and tonsillar stromal cells: Implications for viral latency. J. Virol..

[111] Monaco M.C., Jensen P.N., Hou J., Durham L.C., Major E.O. (1998). Detection of JC virus DNA in human tonsil tissue: Evidence for site of initial viral infection. J. Virol..

[112] Kato A., Kitamura T., Takasaka T., Tominaga T., Ishikawa A., Zheng H., Yogo Y. (2004). Detection of the archetypal regulatory region of JC virus from the tonsil tissue of patients with tonsillitis and tonsillar hypertrophy. J. Neurovirol..

[113] Comar M., Zanotta N., Bovenzi M., Campello C. (2010). JCV/BKV and SV40 viral load in lymphoid tissues of young immunocompetent children from an area of North-East Italy. J. Med. Virol..

[114] Ruggiero F., Carbone D., Mugavero R., Palmieri A., Lauritano D., Baggi L., Nardone M., Martinelli M., Carinci F. (2018). Human polyomavirus in tonsillar microbiota of an Afgan population group. J. Biol. Regul. Homeost. Agents.

[115] Mazzoni E., Pellegrinelli E., Mazziotta C., Lanziollotti C., Rotondo J.C., Bononi I., Iaquinta M.R., Manfrini M., Vesce F., Tognon M. (2020). Mother-to-child transmission of oncogenic polyomaviruses BKPyV, JCPyV and SV40. J. Infect..

[116] Yogo Y., Kitamura T., Sugimoto C., Ueki T., Aso Y., Hara K., Taguchi F. (1990). Isolation of a Possible Archetypal JC Virus DNA Sequence from Nonimmunocompromised Individuals. J. Virol..

[117] Daniel A.M., Swenson J.J., Mayreddy R.P.R., Khalili K., Frisque R.J. (1996). Sequences within the Early and Late Promoters of Archetype JC Virus Restrict Viral DNA Replication and Infectivity. Virology.

[118] McIlroy D., Halary F., Bressollette-Bodin C. (2018). Intra-patient viral evolution in polyomavirus-related diseases. Phil. Trans. R. Soc. B.

[119] Ciardi M.R., Zingaropoli M.A., Iannetta M., Prezioso C., Perri V., Pasculli P., Lichtner M., d’Ettorre G., Altieri M., Conte A. (2020). JCPyV NCCR analysis in PML patients with different risk factors: Exploring common rearrangements as essential changes for neuropathogenesis. Virol. J..

[120] Tominaga T., Yogo Y., Kitamura T., Aso Y. (1992). Persistence of archetypal JC virus DNA in normal renal tissue derived from tumor-bearing patients. Virology.

[121] Jensen P.N., Major E.O. (2001). A classification scheme for human polyomavirus JCV variants based on the nucleotide sequence of the noncoding regulatory region. J. Neurovirol..

[122] Sunyaev S.R., Lugovskoy A., Simon K., Gorelik L. (2009). Adaptive mutations in the JC virus protein capsid are associated with progressive multifocal leukoencephalopathy (PML). PLoS Genet..

[123] Gorelik L., Reid C., Testa M., Brickelmaier M., Bossolasco S., Pazzi A., Bestetti A., Carmillo P., Wilson E., McAuliffe M. (2011). Progressive multifocal leukoencephalopathy (PML) development is associated with mutations in JC virus capsid protein VP1 that change its receptor specificity. J. Infect. Dis..

[124] Marshall L.J., Ferenczy M.W., Daley E.L., Jensen P.N., Ryschkewitsch C.F., Major E.O. (2014). Lymphocyte gene expression and JC virus noncoding control region sequences are linked with the risk of progressive multifocal leukoencephalopathy. J. Virol..

[125] Ray U., Cinque P., Gerevini S., Longo V., Lazzarin A., Schippling S., Martin R., Buck C.B., Pastrana D.V. (2015). JC polyomavirus mutants escape antibody-mediated neutralization. Sci. Transl. Med..

[126] Assetta B., Atwood W.J. (2017). The Biology of JC Polyomavirus. Biol. Chem..

[127] Agostini H.T., Ryschkewitsch C.F., Singer E.J., Stoner G.L. (1997). JC virus regulatory region rearrangements and genotypes in progressive multifocal leukoencephalopathy: Two independent aspects of virus variation. J. Gen. Virol..

[128] Chapagain M.L., Nerurkar V.R. (2010). Human polyomavirus JC (JCV) infection of human B lymphocytes: A possible mechanism for JCV transmigration across the blood-brain barrier. J. Infect. Dis..

[129] Major E.O., Amemiya K., Elder G., Houff S.A. (1990). Glial cells of the human developing brain and B cells of the immune system share a common DNA binding factor for recognition of the regulatory sequences of the human polyomavirus, JCV. J. Neurol. Res..

[130] Rieckmann P., Michel U., Kehrl J.H. (1994). Regulation of JC Virus Expression in B Lymphocytes. J. Virol..

[131] Wei G., Liu C.K., Atwood W.J. (2000). JC Virus binds to primary human glial cells, tonsillar stromal cells, and B-lymphocytes, but not to T lymphocytes. J. Neurovirol..

[132] Banks W.A., Erickson M.A. (2010). The blood-brain barrier and immune function and dysfunction. Neurobiol. Dis..

[133] Haley S.A., O’Hara B.A., Nelson C.D., Brittingham F.L., Henriksen K.J., Stopa E.G., Atwood W.J. (2015). Human polyomavirus receptor distribution in brain parenchyma contrasts with receptor distribution in kidney and choroid plexus. Am. J. Pathol..

[134] O’Hara B.A., Gee G.V., Atwood W.J., Haley S.A. (2018). Susceptibility of Primary Human Choroid Plexus Epithelial Cells and Meningeal Cells to Infection by JC Virus. J. Virol..

[135] Corbridge S.M., Rice R.C., Bean L.A., Wüthrich C., Dang X., Nicholson D.A., Koralnik I.J. (2019). JC virus infection of meningeal and choroid plexus cells in patients with progressive multifocal leukoencephalopathy. J. Neurovirol..

[136] Wharton K.A., Quigley C., Themeles M., Dunstan R.W., Doyle K., Cahir-McFarland E., Wei J., Buko A., Reid C.E., Sun C. (2016). JC Polyomavirus Abundance and Distribution in Progressive Multifocal Leukoencephalopathy (PML) Brain Tissue Implicates Myelin Sheath in Intracerebral Dissemination of Infection. PLoS ONE.

[137] White F.A., Ishaq M., Stoner G.L., Frisque R.J. (1992). JC virus DNA is present in many human brain samples from patients without progressive multifocal leukoencephalopathy. J. Virol..

[138] Lynch K.J., Frisque R.J. (1991). Factors contributing to the restricted DNA replicating activity of JC virus. Virology.

[139] Kim S., Choi E., Jo Y.W., Henson J.W., Kim H. (2004). Transcriptional activation of JC virus early promoter by phorbol ester and interleukin-1β: Critical role of nuclear factor-1. Virology.

[140] White M.K., Safak M., Khalili K. (2009). Regulation of gene expression in primate polyomaviruses. J. Virol..

[141] Kondo Y., Windrem M.S., Zou L., Chandler-Militello D., Schanz S.J., Auvergne R.M., Betstadt S.J., Harrington A.R., Johnson M., Kazarov A. (2014). Human glial chimeric mice reveal astrocytic dependence of JC virus infection. J. Clin. Investig..

[142] Ravichandran V., Major E.O. (2008). DNA-binding transcription factor NF-1A negatively regulates JC virus multiplication. J. Gen. Virol..

[143] Chen X.S., Stehle T., Harrison S.C. (1998). Interaction of polyomavirus internal protein VP2 with the major capsid protein VP1 and implications for participation of VP2 in viral entry. EMBO Rep..

[144] White M.K., Khalili K. (2011). Pathogenesis of progressive multifocal leukoencephalopathy revisited. J. Infect. Dis..

[145] Trowbridge P.W., Frisque R.J. (1995). Identification of three new JC virus proteins generated by alternative splicing of the early viral mRNA. J. Neurovirol..

[146] Seo G.J., Fink L.H., O’Hara B., Atwood W.J., Sullivan C.S. (2008). Evolutionarily conserved function of a viral microRNA. J. Virol..

[147] Swenson J.J., Frisque R.J. (1995). Biochemical characterization and localization of JC virus large T antigen phosphorylation domains. Virology.

[148] Neu U., Maginnis M.S., Palma A.S., Ströh L.J., Nelson C.D., Feizi T., Atwood W.J., Stehle T. (2010). Structure-function analysis of the human JC polyomavirus establishes the LSTc pentasaccharide as a functional receptor motif. Cell Host Microbe.

[149] Ströh L.J., Maginnis M.S., Blaum B.S., Nelson C.D., Neu U., Gee G.V., O’Hara B.A., Motamedi N., DiMaio D., Atwood W.J. (2015). The Greater Affinity of JC Polyomavirus Capsid for α2,6-Linked Lactoseries Tetrasaccharide c than for Other Sialylated Glycans Is a Major Determinant of Infectivity. J. Virol..

[150] Haley S.A., O’Hara B.A., Atwood W.J. (2020). Adipocyte Plasma Membrane Protein (APMAP) promotes JC Virus (JCPyV) Infection in Human Glial Cells. Virology.

[151] Pho M.T., Ashok A., Atwood W.J. (2000). JC virus enters human glial cells by clathrin-dependent receptor-mediated endocytosis. J. Virol..

[152] Querbes W., Benmerah A., Tosoni D., Di Fiore P.P., Atwood W.J. (2004). A JC virus-induced signal is required for infection of glial cells by a clathrin- and eps15-dependent pathway. J. Virol..

[153] Maginnis M.S., Haley S.A., Gee G.V., Atwood W.J. (2010). Role of N-linked glycosylation of the 5-HT2A receptor in JC virus infection. J. Virol..

[154] Assetta B., Maginnis M.S., Gracia Ahufinger I., Haley S.A., Gee G.V., Nelson C.D., O’Hara B.A., Allen Ramdial S.A., Atwood W.J. (2013). 5-HT2 receptors facilitate JC polyomavirus entry. J. Virol..

[155] Querbes W., O’Hara B.A., Williams G., Atwood W.J. (2006). Invasion of host cells by JC virus identifies a novel role for caveolae in endosomal sorting of noncaveolar ligands. J. Virol..

[156] Raote I., Bhattacharya A., Panicker M.M., Chattopadhyay A. (2007). Serotonin 2A (5-HT2A) Receptor Function: Ligand-Dependent Mechanisms and Pathways. Serotonin Receptors in Neurobiology.

[157] Ashok A., Atwood W.J. (2003). Contrasting roles of endosomal pH and the cytoskeleton in infection of human glial cells by JC virus and simian virus 40. J. Virol..

[158] Shishido-Hara Y., Ichinose S., Higuchi K., Hara Y., Yasui K. (2004). Major and minor capsid proteins of human polyomavirus JC cooperatively accumulate to nuclear domain 10 for assembly into virions. J. Virol..

[159] Nelson C.D., Derdowski A., Maginnis M.S., O’Hara B.A., Atwood W.J. (2012). The VP1 subunit of JC polyomavirus recapitulates early events in viral trafficking and is a novel tool to study polyomavirus entry. Virology.

[160] Fanning E., Zhao K. (2009). SV40 DNA replication: From the A gene to a nanomachine. Virology.

[161] Nesper J., Smith R.W.P., Kautz A.R., Sock E., Wegner M., Grummt F., Nasheuer H. (1997). A Cell-Free Replication System for Human Polyomavirus JC DNA. J. Virol..

[162] DeCaprio J.A., Ludlow J.W., Figge J., Shew J., Huang C., Lee W., Marsilio E., Paucha E., Livingston D.M. (1988). SV40 large tumor antigen forms a specific complex with the product of the retinoblastoma susceptibility gene. Cell.

[163] Stubdal H., Zalvide J., Campbell K.S., Schweitzer C., Roberts T.M., DeCaprio J.A. (1997). Inactivation of pRB-Related Proteins p130 and p107 Mediated by the J Domain of Simian Virus 40 Large T Antigen. Mol. Cell. Biol..

[164] Topalis D., Andrei G., Snoeck R. (2013). The large tumor antigen: A “Swiss Army knife” protein possessing the functions required for the polyomavirus life cycle. Antivir. Res..

[165] Meinke G., Phelan P.J., Kalekar R., Shin J., Archambault J., Bohm A., Bullock P.A. (2014). Insights into the Initiation of JC Virus DNA Replication Derived from the Crystal Structure of the T-Antigen Origin Binding Domain. PLoS Pathog..

[166] Lane D.P., Crawford L.V. (1979). T antigen is bound to a host protein in SY40-transformed cells. Nature.

[167] McCormick F., Harlow E. (1980). Association of a murine 53,000-dalton phosphoprotein with simian virus 40 large-T antigen in transformed cells. J. Virol..

[168] McCormick F., Clark R., Harlow E., Tjian R. (1981). SV40 T antigen binds specifically to a cellular 53 K protein in vitro. Nature.

[169] Welcker M., Clurman B.E. (2005). The SV40 large T antigen contains a decoy phosphodegron that mediates its interactions with Fbw7/hCdc4. J. Biol. Chem..

[170] DeCaprio J.A., Garcea R.L. (2013). A cornucopia of human polyomaviruses. Nat. Rev. Microbiol..

[171] Bollag B., Chuke W., Frisque R.J. (1989). Hybrid Genomes of the Polyomaviruses JC Virus, BK Virus, and Simian Virus 40: Identification of Sequences Important for Efficient Transformation. J. Virol..

[172] Cicala C., Avantaggiati M.L., Graessmann A., Rundell K., Levine A.S., Carbone M. (1994). Simian virus 40 small-t antigen stimulates viral DNA replication in permissive monkey cells. J. Virol..

[173] Cho U.S., Morrone S., Sablina A.A., Arroyo J.D., Hahn W.C., Xu W. (2007). Structural Basis of PP2A Inhibition by Small t Antigen. PLoS Biol..

[174] Bollag B., Hofstetter C.A., Reviriego-Mendoza M.M., Frisque R.J. (2010). JC Virus Small t Antigen Binds Phosphatase PP2A and Rb Family Proteins and Is Required for Efficient Viral DNA Replication Activity. PLoS ONE.

[175] Sariyer I.K., Khalili K., Safak M. (2008). Dephosphorylation of JC virus agnoprotein by protein phosphatase 2A: Inhibition by small t antigen. Virology.

[176] Prins C., Frisque R.J. (2001). JC virus T’ proteins encoded by alternatively spliced early mRNAs enhance T antigen-mediated viral DNA replication in human cells. J. Neurovirol..

[177] Erickson K.D., Bouchet-Marquis C., Heiser K., Szomolanyi-Tsuda E., Mishra R., Lamothe B., Hoenger A., Garcea R.L. (2012). Virion Assembly Factories in the Nucleus of Polyomavirus-Infected Cells. PLoS Pathog..

[178] Erickson K.D., Garcea R.L. (2019). Viral replication centers and the DNA damage response in JC virus-infected cells. Virology.

[179] Jul-Larsen Å., Visted T., Karlsen B.O., Rinaldo C.H., Bjerkvig R., Lønning P.E., Bøe S.O. (2004). PML-nuclear bodies accumulate DNA in response to polyomavirus BK and simian virus 40 replication. Exp. Cell Res..

[180] Orba Y., Suzuki T., Makino Y., Kubota K., Tanaka S., Kimura T., Sawa H. (2010). Large T Antigen Promotes JC Virus Replication in G_2_-arrested Cells by Inducing ATM- and ATR-mediated G_2_ Checkpoint Signaling. J. Biol. Chem..

[181] Shishido-Hara Y., Yazawa T., Nagane M., Higuchi K., Abe-Suzuki S., Kurata M., Kitagawa M., Kamma H., Uchihara T. (2014). JC Virus Inclusions in Progressive Multifocal Leukoencephalopathy: Scaffolding Promyelocytic Leukemia Nuclear Bodies Grow with Cell Cycle Transition through an S-to-G2YLike State in Enlarging Oligodendrocyte Nuclei. J. Neuropathol. Exp. Neurol..

[182] Mattern C.F.T., DeLeva A.M. (1968). Observations on polyoma virus filaments. Virology.

[183] Nagashima K., Yamaguchi K., Nakase H., Miyazaki J. (1982). Progressive Multifocal Leukoencephalopathy: A Case Report and Review of the Literature. Pathol. Int..

[184] Suzuki T., Orba Y., Okada Y., Sunden Y., Kimura T., Tanaka S., Nagashima K., Hall W.W., Sawa H. (2010). The Human Polyoma JC Virus Agnoprotein Acts as a Viroporin. PLoS Pathog..

[185] Saribas A.S., White M.K., Safak M. (2012). JC virus agnoprotein enhances large T antigen binding to the origin of viral DNA replication: Evidence for its involvement in viral DNA replication. Virology.

[186] Saribas A.S., Datta P.K., Safak M. (2020). A comprehensive proteomics analysis of JC virus Agnoprotein-interacting proteins: Agnoprotein primarily targets the host proteins with coiled-coil motifs. Virology.

[187] Craigie M., Cicalese S., Sariyer I.K. (2018). Neuroimmune Regulation of JC Virus by Intracellular and Extracellular Agnoprotein. J. Neuroimmune Pharmacol..

[188] Millan M.J., Marin P., Bockaert J., la Cour C.M. (2008). Signaling at G-protein-coupled serotonin receptors: Recent advances and future research directions. Trends Pharmacol. Sci..

[189] Elphick G.F., Querbes W., Jordan J.A., Gee G.V., Eash S., Manley K., Dugan A., Stanifer M., Bhatnagar A., Kroeze W.K. (2004). The human polyomavirus, JCV, uses serotonin receptors to infect cells. Science.

[190] Assetta B., Morris-Love J., Gee G.V., Atkinson A.L., O’Hara B.A., Maginnis M.S., Haley S.A., Atwood W.J. (2019). Genetic and Functional Dissection of the Role of Individual 5-HT_2_ Receptors as Entry Receptors for JC Polyomavirus. Cell Rep..

[191] Mayberry C.L., Soucy A.N., Lajoie C.R., DuShane J.K., Maginnis M.S. (2019). JC Polyomavirus Entry by Clathrin-Mediated Endocytosis Is Driven by β-Arrestin. J. Virol..

[192] Maginnis M.S., Ströh L.J., Gee G.V., O’Hara B.A., Derdowski A., Stehle T., Atwood W.J. (2013). Progressive multifocal leukoencephalopathy-associated mutations in the JC polyomavirus capsid disrupt lactoseries tetrasaccharide c binding. mBio.

[193] Geoghegan E.M., Pastrana D.V., Schowalter R.M., Ray U., Gao W., Ho M., Pauly G.T., Sigano D.M., Kaynor C., Cahir-McFarland E. (2017). Infectious Entry and Neutralization of Pathogenic JC Polyomaviruses. Cell Rep..

[194] O’Hara B.A., Morris-Love J., Gee G.V., Haley S.A., Atwood W.J. (2020). JC Virus infected choroid plexus epithelial cells produce extracellular vesicles that infect glial cells independently of the virus attachment receptor. PLoS Pathog..

[195] Handala L., Blanchard E., Raynal P., Roingeard P., Morel V., Descamps V., Castelain S., Francois C., Duverlie G., Brochot E. (2020). BK Polyomavirus Hijacks Extracellular Vesicales for En Bloc Transmission. J. Virol..

[196] Giannecchini S. (2020). Evidence of the Mechanism by Which Polyomaviruses Exploit the Extracellular Vesicle Delivery System during Infection. Viruses.

[197] Scribano S., Guerrini M., Arvia R., Guasti D., Nardini P., Romagnoli P., Giannecchini S. (2020). Archetype JC polyomavirus DNA associated with extracellular vesicles circulates in human plasma samples. J. Clin. Virol..

[198] Pulliam L., Gupta A. (2015). Modulation of Cellular Function through Immune-Activated Exosomes. DNA Cell Biol..

[199] Muratori C., Cavallin L.E., Krätzel K., Tinari A., De Militio A., Fais S., D’Aloja P., Federico M., Vullo V., Fomina A. (2009). Massive secretion by T cells is caused by HIV Nef in infected cells and by Nef transfer to bystander cells. Cell Host Microbe.

[200] Feng Z., Hensley L., McKnight K.L., Hu F., Madden V., Ping L., Jeong S.H., Walker C., Lanford R.E., Lemon S.M. (2013). A pathogenic picornavirus acquires an envelope by hijacking cellular membranes. Nature.

[201] Kulkarni R., Prasad A. (2017). Exosomes Derived from HIV-1 Infected DCs Mediate Viral trans-Infection via Fibronectin and Galectin-3. Sci. Rep..

[202] Sadeghipour S., Mathias R.A. (2017). Herpesviruses hijack host exosomes for viral pathogenesis. Semin. Cell Dev. Biol..

[203] Raab-Traub N., Dittmer D.P. (2017). Viral effects on the content and function of extracellular vesicles. Nature Rev. Microbiol..

[204] Zhou W., Woodson M., Neupane B., Bai F., Sherman M.B., Choi K.H., Neelakanta G., Sultana H. (2018). Exosomes serve as novel modes of tick-borne flavivirus transmission from arthropod to human cells and facilitates dissemination of viral RNA and proteins to the vertebrate neuronal cells. PLoS Pathog..

[205] Santiana M., Altan-Bonnet N. (2019). Insane in the Membrane: Glial Extracellular Vesicles Transmit Polyomaviruses. mBio.

[206] Morris-Love J., Gee G.V., O’Hara B.A., Assetta B., Atkinson A.L., Dugan A.S., Haley S.A., Atwood W.A. (2019). JC polyomavirus uses Extracellular Vesicles to Infect Target Cells. mBio.

[207] Agnihotri S.P., Wuthrich C., Dang X., Nauen D., Karimi R., Viscidi R., Bord E., Batson S., Troncoso J., Koralnik I.J. (2014). A Fatal Case of JC Virus Meningitis Presenting with Hydrocephalus in a Human Immunodeficiency Virus-Seronegative Patient. Ann. Neurol..

[208] Balusu S., Van Wonterghem E., De Rycke R., Raemdonck K., Stremersch S., Gevaert K., Brkic M., Demeestere D., Vanhooren V., Hendrix A. (2016). Identification of a novel mechanism of blood-brain communication during peripheral inflammation via choroid plexus-derived extracellular vesicles. EMBO Mol. Med..

[209] Trajkovic K., Hsu C., Chiantia S., Rajendran L., Wenzel D., Wieland F., Schwille P., Brugger B., Simons M. (2008). Ceramide triggers budding of exosome vesicles into multivesicular endosomes. Science.

[210] Dinkins M.B., Dasgupta S., Wang G., Zhu G., Bieberich E. (2014). Exosome reduction in vivo is associated with lower amyloid plaque load in the 5XFAD mouse model of Alzheimer’s disease. Neurobiol. Aging.

[211] Jelcic I., Combaluzier B., Jelcic I., Faigle W., Senn L., Reinhart B.J., Ströh L., Nitsch R.M., Stehle T., Sospedra M. (2015). Broadly neutralizing human monoclonal JC polyomavirus VP1-specific antibodies as candidate therapeutics for progressive multifocal leukoencephalopathy. Sci. Transl. Med..

[212] Osterman J.V., Waddell A., Aposhian H.V. (1970). DNA and gene therapy: Uncoating of polyoma pseudovirus in mouse embryo cells. Proc. Natl. Acad. Sci. USA.

[213] Chao C.N., Yang Y.H., Wu M.S., Chou M.C., Fang C.Y., Lin M.C., Tai C.K., Shen C.H., Chen P.L., Chang D. (2018). Gene therapy for human glioblastoma using neurotropic JC virus-like particles as a gene delivery vector. Sci. Rep..

[214] Lin M.C., Wang M., Chou M.C., Chao C.N., Fang C.Y., Chen P.L., Chang D., Shen C.H. (2019). Gene therapy for castration-resistant prostate cancer cells using JC polyomavirus-like particles packaged with a PSA promoter-driven suicide gene. Cancer Gene Ther..

